# Quality of life and treatment adherence in hypertensive patients: systematic review with meta-analysis

**DOI:** 10.1590/S1518-8787.2016050006415

**Published:** 2016-11-24

**Authors:** Ana Célia Caetano de Souza, José Wicto Pereira Borges, Thereza Maria Magalhães Moreira

**Affiliations:** I Unidade de Farmacologia Clínica. Núcleo de Pesquisa e Desenvolvimento de Medicamentos. Universidade Federal do Ceará. Fortaleza, CE, Brasil; II Programa de Graduação em Enfermagem. Programa de Pós-Graduação em Saúde e Comunidade. Campus Amilcar Ferreira Sobral. Universidade Federal do Piauí. Floriano, PI, Brasil; III Programa de Pós-Graduação em Saúde Coletiva. Programa de Pós-Graduação em Cuidados Clínicos em Enfermagem e Saúde. Centro de Ciências da Saúde. Universidade Estadual do Ceará. Fortaleza, CE, Brasil

**Keywords:** Hypertension, Drug Therapy, Antihypertensive Agents, therapeutic use, Medication Adherence, Quality of Life, Meta-Analysis

## Abstract

**OBJECTIVE:**

To verify the effects of antihypertensive treatment (pharmacological and non-pharmacological) on the health-related quality of life of individuals with hypertension.

**METHODS:**

We conducted a systematic review with meta-analysis using the following databases: IBECS, LILACS, SciELO, Medline, Cochrane, Science Direct, Scopus and the Brazilian Capes Theses and Dissertations Database. The statistical analysis was performed using Review Manager, version 5.2. The average difference was used for the summarization of meta-analytic effect by the fixed-effect model. Twenty studies were included.

**RESULTS:**

The summarization of the effect showed an average increase of 2.45 points (95%CI 1.02–3.87; p < 0.0008) in the quality of life of individuals adhering to non-pharmacological treatment for arterial hypertension. Adherence to pharmacological treatment indicated an average increase of 9.24 points (95%CI 8.16–10.33; p < 0.00001) in the quality of life of individuals with arterial hypertension.

**CONCLUSIONS:**

Non-pharmacological treatment improves the overall quality of life and physical domain of people with arterial hypertension. Adherence to pharmacological treatment has a positive impact on the mental and physical domains of patients, as it did on the overall quality of life score.

## RESUMO

**OBJETIVO:**

Verificar os efeitos do tratamento anti-hipertensivo (farmacológico e não-farmacológico) na qualidade de vida relacionada à saúde de pessoas com hipertensão arterial.

**MÉTODOS:**

Foi conduzida revisão sistemática com metanálise utilizando as bases de dados IBECS, Lilacs, SciELO, Medline, Cochrane, Science Direct, Scopus e o banco de teses da Capes. A análise estatística foi realizada pelo *Review Manager* versão 5.2. Foi utilizada a diferença da média na sumarização do efeito metanalítico pelo modelo de efeito fixo. Vinte estudos foram incluídos.

**RESULTADOS:**

A sumarização do efeito mostrou incremento de 2,45 pontos na média (IC95% 1,02–3,87; p < 0,0008) da qualidade de vida em pessoas com adesão ao tratamento não farmacológico para hipertensão arterial. A adesão ao tratamento farmacológico indicou aumento de 9,24 pontos na média (IC95% 8,16–10,33; p < 0,00001) da qualidade de vida em pessoas com hipertensão arterial.

**CONCLUSÕES:**

O tratamento não-farmacológico melhora a qualidade de vida global e o domínio físico de pessoas com hipertensão arterial. A adesão ao tratamento farmacológico impacta positivamente nos domínios mental, físico e escore total da qualidade de vida.

## INTRODUCTION

Cardiovascular diseases are a global public health problem that affects a significant portion of the population^9^ ^,^ ^16^ ^,^ ^33^. There is an increasing rate of mortality as a result of cardiovascular disease among the Brazilian population, with hypertension being one of these conditions, whose estimated prevalence is 35.0% of the population aged older than 40 years^25^. Between 2000 and 2013, the number of deaths associated with hypertension in the United States increased by 61.8%^16^.

The epidemiological impacts of hypertension are undeniable and induce further discussion in the context of healthcare. Recent evidence suggests that hypertension has been a contributing factor for reducing patients’ health-related quality of life (HRQoL) when compared to that of normotensive patients^35^. The quality of life of people with hypertension is affected by several factors, among them are linked to the very existence of infirmity and its chronic-degenerative character, to the discovery of the disease, to the negative effects on physical, emotional and social aspects, as well as those related to medication therapy^5^ ^,^ ^6^. However, studies have shown that antihypertensive can significantly increase blood pressure control, while simultaneously improving patient HRQoL^31^ ^,^ ^37^ and decreasing the frequency of complications resulting from hypertension.

It is possible to predict which patients, while adhering to both their pharmacological and non-pharmacological treatment regimens, are most likely to experience improvements in HRQoL. A study conducted in Spain showed that adhering to pharmacological treatment improves quality of life^19^. A study conducted in the USA found low physical and mental quality of life scores in elderly people who did not adhere to their pharmacological treatment^15^. On the other hand, studies in Pakistan concluded that there is a weak correlation between adhering to hypertension treatment and HRQoL, which indicates that there is an insignificant relationship between them. The Pakistani study suggests that there are other factors responsible for impacting HRQoL during the course of treatment^27^ ^,^ ^28^.

These individual studies give rise to controversy in regards to the presence and significance of the impact of treatment and adherence in HRQoL in people with hypertension. Our study will make it possible to deepen knowledge regarding the association between quality of life and treatment adherence in people with hypertension, while also clarifying the effect of treatment adherence on the HRQoL of patients, given the subject’s still scarce inclusion in global literature. Based on the above, the objective of our study was to verify the effects of adhering to antihypertensive treatment (pharmacological and non-pharmacological) on HRQoL of people with arterial hypertension.

## METHODS

There was a systematic literature review performed with meta-analysis following the recommendations from Cochrane^14^. Searches were conducted on the databases IBECS, LILACS, SciELO, Medline, Cochrane, Science Direct, Scopus and, the *Banco de Teses da Capes* (Brazilian Theses and Dissertations Database) were used and manual searches made in the references of the selected studies as representative of the gray literature. The searches were performed from January to March 2014, with no temporal delimitation placed on the publications.

The research question was elaborated using the PICO strategy: “What are the effects of adhering to antihypertensive treatment (pharmacological and non-pharmacological) on quality of life for people with arterial hypertension?” The decision to include adherence to pharmacological and non-pharmacological treatment in the same meta-analysis was made because the descriptors concerning adherence to the treatment include studies on these two dimensions, and are therefore common for the two types of treatment. Thus, the focus on treatment adherence was maintained and the analytical capacity of the review preserved, since treatment adherence brings these two dimensions together.

The general search expression used was (((((((hypertension[MeSH Terms]) OR blood pressure[MeSH Terms]) AND Patient Adherence[MeSH Terms]) OR Patient Cooperation) OR Medication Adherence[MeSH Terms]) OR Medication Compliance[MeSH Terms]) AND Quality of Life[MeSH Terms]). The term Patient Cooperation was used as a non-controlled descriptor. The search expression underwent adaptations that are required by the specificities of each base.

The inclusion criteria were: observational studies (cohort, case-control) and randomized clinical trials; people with primary hypertension, aged above 18 years; treatment adherence status; quality of life score using validated instruments; languages: Portuguese, English and Spanish.

**Figure 1:**
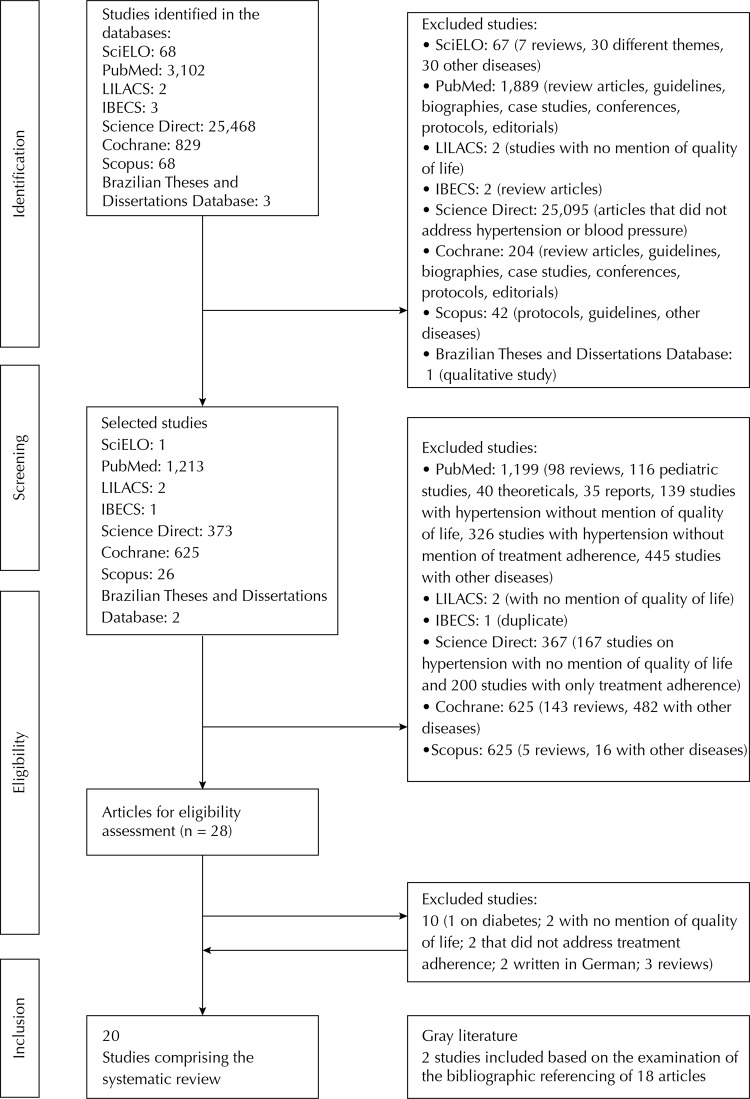
Study selection flowchart. Fortaleza, CE, Northeastern Brazil, 2015.

The PRISM Protocol was used (Preferred Reporting Items for Systematic reviews and Meta-Analyses)^14^ to report the selection of studies. There were initially two independent researchers conducting the search to identify the potential primary studies. This process involved the studies going through three filters for selection and assessment:

First filter (selection of relevant publications): The flow of the procedures and the corresponding numbers in the search and selection process of the research projects are described in Figure 1. By March 30th, 2014, the systematic searches had recovered 29,543 potentially relevant references from eight databases that had been investigated. Based on the databases’s filters, 27,301 studies, which were different than what was specified in our inclusion criteria, were identified and were excluded. Of these articles, 25,095 were from Science Direct, with the other 2,206 articles from seven other databases, showing relative adequacy of search key used in the latter databases.

Due to the volume of publications from *Science Direct,* a refinement filter was used for the database itself. The filter used was ‘*topic’,* which presents the content present in the studies. Adopting a safety margin in this refinement process involved outlining the following strategy: 1) activation of topics that did not designate the scope of systematic review: the topics *cancer*, *pulmonary hypertension, glaucoma, ocular hypertension, AIDS, animal, pain* and *heart failure* were activated. For each topic from the actioned filter, reviewers observed the list of articles to detect possible titles of interest. The only topic that showed subjects that correlated to those of the systematic review was *heart failure*. Following this preliminary procedure, the next step was taken; 2) filter activation of topics that designated the scope of the systematic review: the topics *blood pressure*, *hypertension*, *treatment*, *health care* and *quality of life* were activated, with the listing of the studies being observed based on the activation of each topic, which made it possible to confirm the titles of interest; 3) finally, all the topics of the second step were activated (*blood pressure,* hypertension, *treatment*, *health care* and *quality of life*) in addition to the topic of the first step, *heart failure*, which had already proved to contain studies of interest to the review.

Thus, despite the automatic filters of databases not being trusted to exclude studies, for being outside the reviewers’ control, the adopted procedure made it possible to achieve clarity in the content of the topics and their relationship with the scope of the systematic review, ensuring the safe application of the choice of filters. These procedures enabled a large number of references to be excluded (25,095 articles).

These articles were then referred for evaluation based on title and summary (2,243 studies). Of these articles, 2,215 were then excluded during the screening process (Figure 1). The objective of this step was to disregard irrelevant articles. Each researcher ended up with a list of primary studies. The two lists were compared with each other, with one single list being the result. Any articles that generated disagreement during inclusion or exclusion were also included at that stage.

Twenty-eight eligible studies were read in their entirety, 10 of which were excluded. The 18 selected studies were read once again and their references were manually examined by researchers. Two studies were identified during this process, which were added to those previously selected, resulting in 20 studies included (Table). The main reason excluding studies in the review was for not having the two variables in the core of the article (treatment adherence and quality of life).

**Table:** Characterization of studies on quality of life and treatment adherence in hypertensive individuals. Fortaleza, CE, Northeastern Brazil, 2015.

Authors Year, country	Type of study	Participants			Measurement methods		Intervention	Results	Score NOS^a,c,d^
	
		Average age (years)	Sample	Sampling	Quality of life	Treatment adherence			
Quez et al.^24^ (1988), USA	Quasi-experimental. Hospital-based.	52.5	30 patients	Non-randomized. Treatment with placebo and indapamide.	*“Well being-ill being” Clinical Observation Scale*	Clinical outcome	Administration of indapamide	2.17 point gain on the well-being scale. Average reduction in PA 25.7/16.2 mmHg.	5^c^
Ameling et al.^1^ (1991), Holland	Randomized, double-blind clinical trial. Community-based.	47	Cases: 331. Controls: 137	Randomized. Cases: people with PAD > 95 mm/Hg. Controls: people with PAD < 95 mm/Hg.	*Amsterdam Mood List* (ASL); *Inventory Physical state*	Clinical outcome	Administration of betaxolol	With no difference in quality of life among the groups.	9^d^
Lee et al.^18^ (1992), USA	Randomized, double-blind clinical trial. Community-based	48	620 patients	Randomized. Three groups: 1/3 received captopril, 1/3 methyldopa, 1/3 propranolol.	*The General Well Being Adjustment Scale* (GWB)	Response from the patient	Administration of captopril, methyldopa, propranolol	With no difference in quality of life among the groups.	8^d^
Novo et al.^22^ (1993), Italy	Multicenter trial. Hospital-based.	68.33	125 patients	Non-randomized. 58.9% used captopril and 41.1% captopril with hydrochlorothiazide.	*Quality of Life Questionnaire* (developed by the authors)	Clinical outcome	Administration of captopril HCTZ	Improvement in all quality of life parameters except libido.	6^c^
Testa et al.^34^ (1993), USA	Randomized multicenter clinical trial. Hospital-based.	64.4	Cases: 192. Controls: 187	Randomized. Cases: received captopril. Controls: received enalapril.	*Scales: Psychological Well-being; General Perceived Health*	Clinical outcome	Administration of captopril and Enalapril	0.11 unit gain in quality of life total with captopril. 0.11 unit loss in the group with enalapril, leading to negative changes in sexual function.	9^d^
McCorvey et al.^20^ (1993), USA	Randomized, double-blind clinical trial. Community-based.	66	17 patients	Randomized. 17 patients who used placebos and antihypertensive agents.	*The Nottingham Health Profile*	Tablet count	Administration of HCTZ, propranolol and enalapril	Difficulties sleeping were frequent when using antihypertensive medications compared with placebo, which were greater when using propranolol.	7^d^
Paran, Anson, Neumann^23^ (1996), USA	Randomized, non-blinded clinical trial. Hospital-based.	54	Cases: 53 Controls: 80	Randomized. Cases: treated with captopril. Controls: treated with beta-blockers.	*Quality of Life Questionnaire* (developed by the authors)	Clinical outcome	Administration of captopril	Improved psychological well-being in both groups. Decline in social activities and perceptions on health deteriorated in the control group.	8^d^
Cleophas et al.^8^ (1997), Netherlands	Randomized, double-blinded multicenter clinical trial. Hospital-based.	58.6	Cases: 70 Controls: 62	Randomized. Cases: use of celiprolol. Controls: use of atenolol.	*The Bulpitt and Flecher Questionnaire of Quality of Life*	Tablet count	Administration of celiprolol and atenolol for 24 weeks	Worsening in sexual function for both groups, which was more pronounced in those using atenolol. Atenolol made patients less alert, requiring more hours of sleep.	8^d^
Barón-Riviera et al.^2^ (1998), Mexico	Randomized clinical trial. Community-based.	51.8	Cases: 68 Controls: 71	Randomized. Cases: educational intervention. Controls: standard consultation.	*Índice de cambio en la calidad de vida*	Clinical outcome, response by patient	Educational intervention on hypertension, nutrition and physical activity	There were changes in the perception of quality of life and sexual functioning in the case group. The degree of improvement in physical strength and mood was higher in the experimental group.	7^d^
Vivian^36^ (2002), USA	Randomized clinical trial. Hospital-based.	64.7	Cases: 26 Controls: 27	Randomized. Cases: medical care + pharmaceutical care. Controls: medical care.	SF-36 *Short-Form Health Survey*	Tablet count and patient response	Pharmaceutical care management	Patients in the control group reported greater bodily pain scores compared to the case group.	7^d^
Cotê, Farris, Feeny^10^ (2003), Canada	Longitudinal. Community-based.	65.6	664 patients	Cases: 100 hypertensive patients. Controls: 199 high-risk individuals and 365 elderly patients.	SF-12 *Short Form Health survey*	*Morisky’s instrument*	-	Physical and mental components were positively correlated with treatment adherence.	7c
Dahlöf et al.^11^ (2005), USA	Randomized, double-blind clinical trial. Hospital-based.	51.6	Cases: 300 Controls: 545	Randomized. Cases: use of felodipine + metoprolol. Controls: use of enalapril or placebo.	*The Psychological General Well-being Index* (PGWB)	Clinical outcome	Administration of felodipine + metropolol or enalapril or placebo	The total average quality of life scores were relatively high in all treatment groups at the beginning of the study, and remained relatively constant throughout the study.	9d
Mohammadi et al.^21^ (2006), Iran	Randomized clinical trial. Hospital-based.	50	Cases: 75 Controls: 75	1 Health Center was selected to be the case and another to be the control group.	SF-36 *Short-Form Health Survey*	*Questionnaire for measuring the patients compliance*	Educational program (nature, causes and complications of hypertension)	Increase of 4 points on quality of life following intervention in the case group and a loss of 2 points in the control group.	8^d^
Schulz et al.^30^(2008), USA	Randomized clinical trial. Community-based.	58.23	440 patients	Allocation into three groups: low social support (n=169); average social support (n=143); and high social support (n=128).	SF-36 *Short-Form Health Survey*	Response from the patient	Program for lifestyle change	Best quality of life in the vitality and physical health subscales. High attendance at social support group improves quality of life.	8^d^
Schmidt et al.^29^ (2008), Germany	Prospective multicenter observational study. Community-based.	60.5	Cases: 4,252 Controls: 2,805 with chronic disease and 610 with hypertension	Non-randomized. Cases: hypertensive patients with olmesartan. Controls: patients with chronic disease; and high blood pressure.	SF-12 *Short-Form Health Survey*	Clinical outcome	Administration of olmesartan	After 6 weeks of therapy with olmesartan, the physical component of quality of life improved compared with the untreated group. The mental component showed no difference among the groups.	7^d^
Bramlage et al.^4^ (2010), Germany	Prospective multicenter observational study. Community-based.	62.8	Cases: 8,237 Controls: 3,415	Cases: treated with olmesartan+amlodipine. Controls: two distinct databases.	SF-12 *Short-Form Health Survey*	Clinical outcome	Administration of Olmesartan + amlodipine	After 18 weeks of therapy with olmesartan+amlodipine, the scores for the physical and mental component were higher than the control group.	7^d^
Lima^b^ (2012), Brazil	Prospective observational study. Community-based	61.7	295 patients	Hypertensive patients at two basic health units.	SF-36 *Short-Form Health Survey*	Clinical outcome	-	People who adhered to treatment had worse HRQoL results compared to those that did not	5^c^
Luriziére et al.^17^ (2013), Canada	Quasi-experimental. Community-based.	54.15	Cases: 21 Controls: 19	Cases: even numbers in the order of arrival. Cases: odd numbers in the order of arrival.	SF-36 *Short-Form Health Survey*	Response from the patient	Educational program	Improvement in the quality of life of the group if in the first month of intervention.	8^d^
Carvalho Siqueira, Sousa^7^ (2013), Brazil	Observational case control. Community-based	61.5	Cases: 246 Controls: 87	Randomized. Cases: hypertensive individuals. Controls: normotensive individuals.	SF-36 *Short Form Health survey*	Clinical outcome	-	The diagnosis time interfered with the physical and mental aspects. Neither the controlled or uncontrolled PA interfered with quality of life.	8^d^
Zyould et al.^37^ (2013), Palestine	Prospective observational study. Hospital-based.	58.4	410 patients	Systematic allocation into three groups: low adherence rate (n=151), average adherence rate (n=110), high adherence rate (n=149).	*European Quality of life Scale* (EQ-5D-5L) and EQ *Visual Analogue Scale* (EQ-VAS)	*Morisky eight-item Medication Adherence Scale* (MMAS)	-	Patients with a high adherence rate had high quality of life values compared to those with a low or average adherence rate.	6^d^

DBP: diastolic blood pressure; BP: blood pressure; HCTZ: hydrochlorothiazide

^a^ Wells G et al. The Newcastle-Ottawa scale (NOS) for assessing the quality of nonrandomised studies in meta-analysis. 2011 [see footnote].

^b^ Lima RA. Fatores que influenciam a qualidade de vida de pacientes hipertensos [dissetation]. São Luís; 2012 [see footnote].

^d^ Newcastle - Ottawa Quality Assessment Scale Case Control Studies.

^c^ Newcastle - Ottawa Quality Assessment Scale Cohort Studies.

The reference lists from the selected studies were examined. The abstracts from the titles that were identified as being linked to the starting question were retrieved and analyzed. The full text was extracted for those abstracts in which a link to the starting question was confirmed, comparing it with the inclusion and exclusion criteria, so that each article could be included in the systematic review.

Second filter (publication selections based on the quality criteria): all the selected studies were accurately examined based on the inclusion and exclusion criteria by two researchers, the objective of which to confirm the included studies. Evaluating the quality of the studies involved using the Newcastle-Ottawa Scale instrument^a^.

This step involved two independent researchers evaluating the studies selected during the first filter. Consensus was reached following the evaluation. Conflicting data were resolved based on the elements of the protocol, which promoted greater accuracy and avoided bias. The inter-raters agreement in the study was confirmed by a 0.87 Kappa coefficient.

Third filter (selection of relevant data): Selecting the relevant data involved using two distinct formulas, one for the case-control or clinical trial studies, and another for the cohort studies. The following information was collected: title, authors, researchers who conducted the evaluation, study type, evaluation date, source, place of publication, publication date, inclusion criteria, sample, quality evaluation of the studies, the quality of life scores and treatment adherence.

The data were organized^14^ into subgroups that had a common focus: (i) adherence to pharmacological treatment; and (ii) adherence to non-pharmacological treatment. Their effects on the mental component, the physical component and the total HRQoL score were subsequently analyzed, which enabled the similarities (homogeneities) and the differences (heterogeneities) between them to be detected.

The meta-analysis required the existence of at least two studies that answer the same question, use at least one common outcome and have similar designs^14^. The statistical analysis was performed using the Review Manager version 5.2 (The Nordic Cochrane Centre, Copenhagen, Denmark).

The homogeneity investigation determined the choice of the meta-analytic model. The random effects model was chosen when the heterogeneity was between moderate and high, according to the Cochran’s Q and statistics I Tests^2^. The fixed effects model was used in instances where there was low or no heterogeneity. For continuous variables, the average difference was used for the summarization of effect; for the dichotomous variables, we used the odds ratio and applied the Mantel-Haenszel method. A 95%CI was considered in both cases (p < 0.05)^26^.

It is important to note that, in cases where there are very small study subgroups, despite the random effects model being preferable, the consideration of authors, which guide the implementation of the fixed-effects model, were followed to obtain an estimate of variance among the studies with a good level of accuracy^3^ ^,^ ^13^ ^,^ ^26^.

The data were presented in forest plot graphs. The publication bias was evaluated by inspection of the funnel plot.

## RESULTS

### Characteristics of the Studies Included

There were distinct tools used to assess the quality of life, however the questionnaire SF-36 and its short version, SF-12, were the most frequently used (42.0% of the studies). Over half (52.6%) of the studies used scales regarding general well-being, psychological, and physical stress, as well as visual analog scales to evaluate quality of life. Only one study (5.2%) used a specific instrument to measure quality of life in people with hypertension. Treatment adherence was measured by the clinical outcome indicated by the reduction or control of systolic and diastolic blood pressure, and by the patients’ responses to individual questions. The Morisky instrument was used in two studies (10.5%) and counting tablets was used in three (15.7%) (Table).

Analyzing 20 studies made it possible to organize the data into two subgroups: one group with studies on adherence to non-pharmacological treatment and quality of life, using educational programs, educational interventions and health education initiatives for promoting quality of life in people with hypertension (five studies), and the other with studies that established a relationship between quality of life and adherence to antihypertensive pharmacological treatment. Each sub-group contained mental dimension, physical dimension and total quality of life score variables.

### Quality Evaluation

In this study, the inter-rater Kappa coefficient was 0.87% for applying the Newcastle-Ottawa Scale^a^ in the selected studies. The minimum score attributed was five stars, with the maximum being nine. In regards to selection, three stars were awarded for the vast majority of the studies (72.2%). In relation to comparability, the vast majority of studies (72.2%) were given a score of two stars. For exhibition, more than half of the studies (65.0%) reached a score of three or more stars. All the studies were generally of high scientific quality (Table).

### Meta-analysis

**Figure 2:**
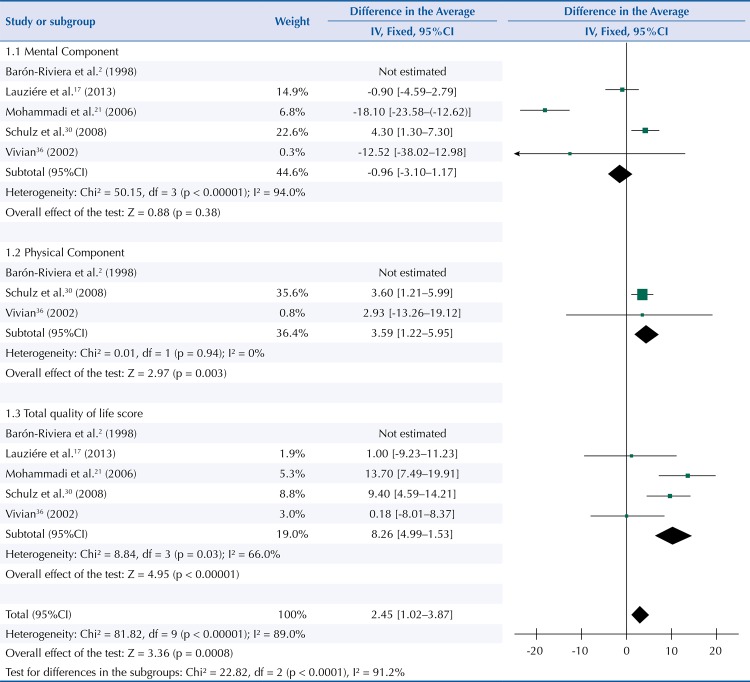
Mean differences in mental, physical components and total quality of life score in hypertensive patients adhering to non-pharmacological treatment. Fortaleza, CE, Northeastern Brazil, 2015.

Five studies evaluated quality of life in people with adherence to non-pharmacological treatment. The data from these studies were assessed using the fixed effects model. Adherence to non-pharmacological treatment, such as participation in educational interventions, showed no positive association with the mental aspect of quality of life (average score =-0.96; 95%CI -3.10–1.17; p = 0.38) (Figure 2). However, in the physical aspect, there was an average increase of 3.59 (95%CI 1.22–5.95; p = 0.003) (Figure 2). Analyzing the total quality of life score showed an average increase of 8.26 (95%CI 4.99–11.53; p < 0.00001) points for the individuals (Figure 2). The summarization of the effect showed an average increase of 2.45 points (95%CI 1.02–3.87; p <0.0008) for the quality of life in people who adhered to pharmacological treatment in the form of educational interventions.

We feel it is worth emphasizing that one study was removed for these analyses^2^, because it caused bias in the result, based on the analysis of the funnel plot. The qualitative analysis indicated heterogeneity due to the difference in quality of life scores used, which culminated in the article^2^ being disregarded from the meta-analytical calculation.

**Figure 3:**
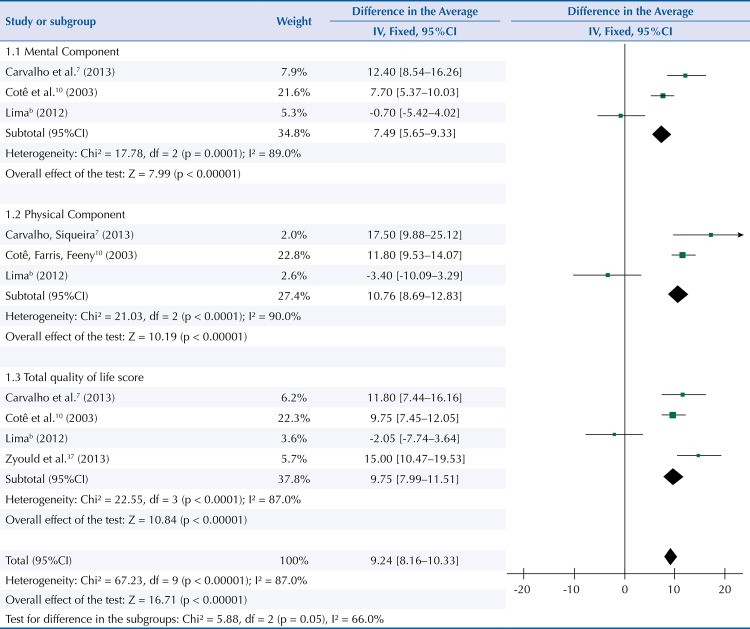
Mean differences in mental, physical components and total quality of life score in hypertensive patients adhering to pharmacological treatment. Fortaleza, CE, Northeastern Brazil, 2015.

Figure 3 shows the mean differences in mental and physical components as well as the total quality of life score in hypertensive patients adhering to pharmacological treatment. These patients showed an average increase of 7.49 (95%CI 5.65–9.33; p < 0.00001) in the mental aspect of quality of life. In the physical aspect, the increase was 10.76 points (95%CI 8.69–12.83; p < 0.00001). The impact of adherence to pharmacological treatment on the total quality of life score was averagely 9.75 points (95%CI 7.99–11.51; p = 0.00001). Due to the small number of studies that provided the meta-analysis with data regarding adherence to pharmacological treatment and quality of life, the fixed effects model was used. The summarization of the effect indicated an average increase of 9.24 points (95%CI 8.16–10.33; p < 0.00001) for quality of life in people adhering to arterial hypertension treatment.

### Publication bias

The completion and inspection of the funnel plot suggested that there was a publication bias. Egger and Begg^12^ tests were not conducted to detect publication bias as the possible meta-analyses involved a small number of articles and these tests have low statistical capacity for relatively small samples (< 20 studies)^12^, i.e., their predictive accuracy would be restricted. In addition, there is a tendency for researchers to preferentially report statistically significant associations, which, in small samples, would favor the verification of publication bias. However, correcting the meta-analysis for publication bias is generally insufficient to remove such bias, which requires that the examples of bias are explored in the text^12^ ^,^ ^32^, a procedure that was adopted in our study.

## DISCUSSION

The main findings of this systematic review with meta-analysis were: non-pharmacological treatment improves overall quality of life and the physical domain of people with hypertension; and, adherence to pharmacological treatment has positive impacts on the mental and physical domain, as well as on the overall HRQoL score.

A randomized clinical trial conducted in Mexico^2^ demonstrated the effect of non-pharmacological treatment (intervention) on the quality of life for people with hypertension, which was measured using the index of change on quality of life. Form the eight evaluated HRQoL areas, there were only significant differences among the groups in the state of mind aspect (p < 0.001). However, an educational program for pharmaceutical care management conducted in the United States found no significant changes in the six domains of the evaluated SF-36, neither between the intervention or comparison groups at the end of the study (p > 0.2)^36^.

A study conducted in Iran^21^ found an improvement in the quality of life (SF-36) of patients who participated in the educational model, the model for care of partners, reaching an average total HRQoL score of 64 points, compared to those who only received conventional care (average of 50.3 points; p < 0.05). Whereas in the United States^30^, a clinical trial conducted with patients involved in the Multicenter Lifestyle Demonstration Project, which offered social support groups for a year as an educational care measure, indicated that patients who received more than 78.0% of care in these groups improved their HRQoL. The average scores of these groups in the mental aspect was 53.6 and, in the physical was 52.8, compared to those who received less than 78.0% care (41.9 and 49.2 in the mental and physical areas, respectively), however, these were not significant.

The effect of an educational program regarding hypertension held in Canada^17^ showed an improvement in quality of life in the first month of study in an group intervention, with an increase of 2.4 in the scores (95%CI 0.09–4.7; p < 0.04) compared to the regular care group. However, there was no observed improvement in the HRQoL at the end of six months of participation in the intervention group.

The studies^2^ ^,^ ^17^ ^,^ ^21^ ^,^ ^30^ ^,^ ^36^ which refer to adherence to pharmacological treatment as a promoting factor for HRQoL highlight that educational interventions can promote increased scores in its main aspects (physical, mental, sexual function, sleep, among others). Despite this increase not having been significant in some studies^2^ ^,^ ^17^ ^,^ ^36^, the interventions can act as important triggers leading to important reflections in people with hypertension, resulting in them pursuing health behaviors or actions that promote improvement in their quality of life.

Using educational interventions that provide increased awareness and involvement for people to care/treat this disease, while understanding the social, economic and cultural context in which they are exist, can result in an improvement in the HRQoL of those involved and, in addition, in changes in the current epidemiological framework of hypertension and its associated comorbidities.

Adherence to the pharmacological treatment in turn can result in improvements in the mental, physical aspects, as well as in the total HRQoL score^4^ ^,^ ^8^ ^,^ ^11^ ^,^ ^23^ ^,^ ^24^ ^,^ ^29^ ^,^ ^34^. The observation was that the results highlighted an improvement in HRQoL in these groups, which were clear in the physical and mental aspects, or in the total quality of life score, with positive effect on the lives of people with hypertension.

The quality of life of people adhering to antihypertensive treatment varied according to the type of medication used in these studies. There was one quasi-experimental study^24^ that analyzed quality of life based on visual analog scale for overall well-being, which was performed on patients using indapamide and demonstrated an improvement in the HRQOL total score and the mental aspect (p < 0.01). During a multicenter trial^34^ performed on men in the United States, patients treated with captopril showed more favorable changes on overall quality of life, perception, general health, vitality and health status, sleep and emotional control when compared to those treated with enalapril. The initial score in quality of life scale was a significant predictor for change (p < 0.001) in the multivariate and univariate analyses; i.e., regardless of whether captopril or enalapril was used, patients who initial had a lower quality of life prior to using the medication showed an improvement in quality of life following their use of the medication. Those who had better quality of life at the beginning remained the same or had a reduced HRQoL.

Effects of substituting β-blockers for angiotensin-converting-enzyme inhibitor (captopril) on HRQOL were evaluated in a randomized clinical trial conducted in Israel^23^. As for the physical domain, there was an improvement in sleep quality (p < 0.001) and gastrointestinal symptoms (p < 0.001) in patients treated with captopril. In the United States^8^, treatment adherence with two β-blockers (celiprolol and atenolol) suggested a significant improvement (p < 0.05) in four of the five physical symptoms (headache, morning headache, blurred vision, skin flushing and dizziness) evaluated for the quality of life. Blurred vision was the only symptom that did not improve from using atenolol, as was true for skin flushing from using celiprolol. Patients were more satisfied with their lives and less hampered by their blood pressure in both groups.

Treatment adherence in patients using felodipine, enalapril or placebos in a clinical trial conducted in the United States^11^ showed similar quality of life scores among the groups. The average HRQoL score was similar and relatively high in all three treatment groups at the beginning of the study, and remained constant throughout its duration.

Two prospective, non-interventional, multicenter studies performed in Germany evaluated HRQoL with adherence to olmesartan^29^ and olmesartan combined with amlodipine^4^. All the items In the first study from the SF-12 improved significantly during the six-week observational period with olmesartan treatment. The summary of the physical (45.6) and mental (50.8) component both showed improvement (p < 0.01)^29^. In the second study, the 12 items from the SF-12 also improved during the 12-18 week observational period with treatment. The summary of the physical (46.9) and mental (52.4) component both showed improvement (p < 0.0001)^4^.

However, some studies highlight side effects from antihypertensive drugs that have a negative effect on HRQoL. Regular use of methyldopa, captopril or propranolol and betaxolol was investigated during studies conducted in the United States^18^ and Holland^1^. The researchers observed that there was a similar quality of life among the patients, regardless of which of these four medications were used. A placebo-controlled clinical trial conducted in the United States^20^ showed that, for the six evaluated HRQoL aspects (pain, physical mobility, sleep, emotional response, social isolation and energy), the worst evaluation was experienced in the group treated with propranolol, when compared with groups treated with hydrochlorothiazide or enalapril. Compared with the placebo treatment, the other three treatment groups showed an increase in their quality of life scores. In another study^11^, using felodipine combined with metoprolol increased scores for gastrointestinal symptoms, thereby worsening the HRQoL; however, the authors observed a reduction in gastrointestinal symptoms when the patients used enalapril.

Despite some studies highlighting the negative effects of antihypertensive medication on HRQoL, adherence to antihypertensive pharmacological treatment^11^ ^,^ ^18^ ^,^ ^20^ is effecting at sparing people from hypertensive complications, which guarantees an increased life expectancy and improvement in the general well-being of these people.

As for the relationship between quality of life and treatment adherence, one multicenter trial conducted in Italy^22^ found that adherence to pharmacological treatment promoted a statistically significant improvement (p < 0.005) in seven of the eight HRQoL parameters (insomnia, headache, drowsiness, well-being, normal libido, normal physical activity, anxiety and mood). Only the normal libido parameter was not statistically significant. However, one longitudinal study^10^ showed that there were weak correlations observed between adherence to pharmacological treatment and HRQoL in a hypertensive group of patients.

One cross-sectional study performed in Palestine^37^ showed that patients with a higher rate of medication adherence, evaluated by the Morisky Medication Adherence Scale (MMAS), presented high values on the quality of life index scale (EQ-5 d) compared to those with low or average adherence (p < 0.05). One research project conducted in Brazil found similar results, in which people who adhered to the pharmacological treatment reached a higher average, when compared to patients who did not adhere to their medication (p < 0.05)^7^. However, there was one Brazilian study that found that patients who adhered to pharmacological treatment had lower quality of life averages compared those who did not adhere to treatment^b^.

Those studies^7^ ^,^ ^10^ ^,^ ^37^ ^,^ ^b^ that referred to a relationship between quality of life and adherence to pharmacological treatment observed statistically significant associations, namely, there were greater increases in HRQoL when antihypertensive pharmacological treatment was adhered to.

One limitation of this systematic review is in relation to the small number of studies that could be used in the meta-analysis, both in the pharmacological and non-pharmacological treatment, making it viable only the fixed effects model was applied, which discourages the generalization of results.

The suggestion is to develop more intervention studies that touch on promoting adherence to pharmacological and non-pharmacological treatment and their impact on quality of life, because, as was noted in our study, there are positive effects to be had when adhering to hypertension treatment in a number of areas that contribute to quality of life, leaving only to improve how this effect is measured.

Implementing innovative strategies, by associating educational, individual and collective interventions, and using subject-centered care practices for managing their medication intake, can promote improvement in HRQoL.

## Footnotes

a Wells G, Shea B, O’Connell J, Robertson J, Peterson V, Welch V et al. The Newcastle-Ottawa scale (NOS) for assessing the quality of nonrandomized studies in meta-analysis. 2011 [cited 2013 Apr 13]. Available at: http://www.ohri.ca/programs/clinical_epidemiology/oxfordasp

b Lima RA. Fatores que influenciam a qualidade de vida de pacientes hipertensos [dissertation]. São Luís (MA): Universidade Federal do Maranhão; 2012.
